# Role of PRDM1 in Tumor Immunity and Drug Response: A Pan-Cancer Analysis

**DOI:** 10.3389/fphar.2020.593195

**Published:** 2020-12-15

**Authors:** Lujun Shen, Qifeng Chen, Changsheng Yang, Ying Wu, Hui Yuan, Shuanggang Chen, Shunling Ou, Yiquan Jiang, Tao Huang, Liangru Ke, Jinqing Mo, Ziqing Feng, Penghui Zhou, Weijun Fan

**Affiliations:** ^1^ Department of Minimally Invasive Interventional Therapy, Sun Yat-sen University Cancer Center, Guangzhou, China; ^2^ State Key Laboratory of Oncology in South China, Collaborative Innovation Center of Cancer Medicine, Sun Yat-sen University, Guangzhou, China; ^3^ Department of Spine Surgery, Third Affiliated Hospital of Southern Medical University, Guangzhou, China; ^4^ Department of Radiology, Sun Yat-sen University Cancer Center, Guangzhou, China; ^5^ Zhong Shan Medical School, Sun Yat-sen University, M, China

**Keywords:** pan-cancer, PR domain zinc finger protein 1, prognosis, tumor immunity, drug response

## Abstract

**Background:** PR domain zinc finger protein 1 (PRDM1) is a regulator of both B cell and T cell differentiation and plays a critical role in immunosuppression. Its role in tumor immunity and correlation with drug response remain unknown.

**Methods:** This work comprehensively analyzed the transcriptional expression pattern of the PRDM1 among 33 types of malignancies from The Cancer Genome Atlas and the Genotype-Tissue Expression projects. Besides, correlation of the PRDM1 with cancer prognosis, immune infiltrates, checkpoint markers, cancer stemness and drug response were explored.

**Results:** High expression level of PRDM1 were observed in ACC, COAD, LAML, LGG, LUAD, OV, PAAD, STAD, TGCT. Cox regression model showed high expression of PRDM1 in tumor samples correlates with poor prognosis in LGG, PAAD, UVM while favorable prognosis in KIRC, SKCM and THCA. PRDM1 expression positively correlates with the expression of LAG3, CTLA4, PDCD1 (PD-1), CD274 (PD-L1), PDCD1LG2 (PD-L2), TIGIT in the majority of 33 cancer types. PRDM1 positively correlated with TNFRSF14 in LGG and UVM among cancers with unfavorable prognosis; this correlation were weak or even negative in cancers with favorable prognosis. The top negatively enriched KEGG terms in high PRDM1 subgroup were B cell receptor signaling, T cell receptor signaling, and the top negatively enriched HALLMARK terms included IL-2-STAT5 signaling and allograft rejection. The expression of PRDM1 was found positively correlated with cancer stemness in CHOL, KIRP, TGCT, THYM and UVM. A series of targeted drugs and small-molecule drugs with promising efficacy predicted by PRDM1 level were identified.

**Conclusion:** The clinical significance and biological impact of high transcriptional expression of PRDM1 differs across different cancers. Inhibiting the PRDM1-dependent signaling could be a novel and promising strategy of immunotherapy in cancers including LGG, PAAD and UVM.

## Introduction

PR domain zinc finger protein 1 (PRDM1)/B lymphocyte-induced maturation protein 1 (BLIMP1) is a regulator of both B cell and T cell differentiation and plays a critical role in T cell-mediated immunosuppression (Martins et al., 2006; Nutt et al., 2007; Fu et al., 2017). Expression of PRDM1 is essential for the production of interleukin (IL)-10 by a subset of Foxp3+ regulatory T cells with an effector phenotype. Recent studies showed PRDM1 coordinates with other transcription factors to regulate the expression of IL-2, IL-21 and IL-10 in effector T lymphocytes (Rutz and Ouyang, 2016; Fu et al., 2017) and contribute to the overexpression of co-inhibitory receptors, such as CTLA-4 and PD-1 (Chihara et al., 2018). On the other side, there is growing evidences showing that the expression of PRDM1 pose differential prognostic impact across different cancers (Wang et al., 2013; Liu et al., 2017; Zhu et al., 2017; Liu et al., 2018). A comprehensive understanding of the prognostic and immunological impact of PRDM1 among cancer types is essential to develop novel immunotherapies.

The advent of next‐generation sequencing (NGS) and large-scale genomics has enabled oncological research to move beyond single gene analysis to pan-cancer analysis. The Cancer Genome Atlas (TCGA) was established based on the data of different cancers at epigenomic, genomic, proteomic and transcriptomic levels to identify the similarities and differences of vital biological processes in diverse cancers (Tomczak et al., 2015). Therefore, we conducted a pan‐cancer genomic analysis of PRDM1 across 33 cancer types using large‐scale RNA‐sequencing (RNA‐seq) data from TCGA. The aims of this study were to (a) characterize the expression of PRDM1 across different cancer types; (b) assess the prognostic values of PRDM1 across different tumors; (c) evaluate the associations between PRDM1 and tumor immunity features including intratumoral immune infiltrates, checkpoint markers, tumor mutation burden (TMB), microsatellite instability (MSI), which have been identified as potential biomarkers for predicting response to immune checkpoint inhibitor treatment, and (d) evaluate its correlations with drugs response.

## Methods

### Datasets of Patients

The TCGA level 3 RNA sequencing processed data of the 33 types of cancers and the corresponding clinical annotations were acquired using the UCSC cancer genome browser (https://tcga.xenahubs.net, accessed April 2020). As the data on RNA seq of normal tissue is relatively scarce in the TCGA database, the transcriptome data of human normal tissues were additionally acquired on the Genotype Tissue Expression database (GTEx; https://www.gtexportal.org/) to compare the expression level of PRDM1 between tumor and normal samples. The approval from the Ethics Committee was exempted as only the open-access data were used.

Collectively, samples of 33 cancer types (*N* = 10,327) were investigated in the final analysis, including Adrenocortical carcinoma (ACC, *n* = 79), Bladder Urothelial Carcinoma (BLCA, *n* = 411), Breast invasive carcinoma (BRCA, *n* = 1,104), Cervical squamous cell carcinoma and endocervical adenocarcinoma (CESC, *n* = 306), Cholangiocarcinoma (CHOL, *n* = 36), Colon adenocarcinoma (COAD, *n* = 471), Lymphoid Neoplasm Diffuse Large B-cell Lymphoma (DLBC, *n* = 48), Esophageal carcinoma (ESCA, *n* = 162), Glioblastoma multiforme (GBM, *n* = 168), Head and Neck squamous cell carcinoma (HNSC, *n* = 502), Kidney Chromophobe (KICH, *n* = 65), Kidney renal clear cell carcinoma (KIRC, *n* = 535), Kidney renal papillary cell carcinoma (KIRP, *n* = 289), Acute Myeloid Leukemia (LAML, *n* = 151), Brain Lower Grade Glioma (LGG, *n* = 529), Liver hepatocellular carcinoma (LIHC, *n* = 374), Lung adenocarcinoma (LUAD, *n* = 526), Lung squamous cell carcinoma (LUSC, *n* = 501), Mesothelioma (MESO, *n* = 86), Ovarian serous cystadenocarcinoma (OV, *n* = 379), Pancreatic adenocarcinoma (PAAD, *n* = 178), Pheochromocytoma and Paraganglioma (PCPG, *n* = 183), Prostate adenocarcinoma (PRAD, *n* = 499), Rectum adenocarcinoma (READ, *n* = 167), Sarcoma (SARC, *n* = 263), Skin Cutaneous Melanoma (SKCM, *n* = 471), Stomach adenocarcinoma (STAD, *n* = 375), Testicular Germ Cell Tumors (TGCT, *n* = 156), Thyroid carcinoma (THCA, *n* = 510), Thymoma (THYM, *n* = 119), Uterine Corpus Endometrial Carcinoma (UCEC, *n* = 548), Uterine Carcinosarcoma (UCS, *n* = 56), Uveal Melanoma (UVM, *n* = 80).

### PRDM1 Survival-Associated Cancers

Data regarding the gene expression profiles of PRDM1 were extracted from the 33 cancer types in TCGA to form an expression matrix, which is further merged with corresponding clinical information based on patient ID. The Kaplan-Meier (KM) analysis by log-rank test was used to compare the overall survival (OS) for patients stratified based on the median gene expression level of PRDM1. Univariate Cox model was applied in calculating the associations between gene expression levels and patient survival across the 33 cancer types, and a *p* < 0.05 for the PRDM1 in a specific cancer indicated statistical significance.

### PRDM1 and Tumor Immune Microenvironment

The immune infiltrates among different types of cancers was estimated by Tumor Immune Estimation Resource (TIMER, https://cistrome.shinyapps.io/timer/) (Li et al., 2017) and CIBERSORT (Newman et al., 2019), respectively. The associations of PRDM1 expression with each immune infiltrate across the 33 cancer types were analyzed.

Estimation of STromal and Immune cells in MAlignant Tumor tissues using Expression data (ESTIMATE) is an approach that uses gene expression profiles to predict tumor purity and the infiltrating stromal cells/immunocytes in tumor tissues (Becht et al., 2016). The ESTIMATE algorithm produces three scores based on the GSEA of single samples, including 1) stromal score, which determines stromal cells in tumor tissues, 2) immune score, which stands for the immunocyte infiltration level in tumor tissues, and 3) estimate score, which infers the tumor purity. In this study, we used the ESTIMATE algorithm to estimate the immune and stromal scores in tumor tissues according to corresponding transcriptional data and calculated the correlations of these scores with the expression of PRDM1.

The associations of the expression level of PRDM1 with the gene markers in tumor infiltrating immunocytes selected with reference to previous research were further conducted (Cristescu et al., 2018; Pan et al., 2019). The estimated statistical significance and Spearman’s correlation coefficient were generated through correlation analysis. The somatic mutation data of all TCGA patients were downloaded (https://tcga.xenahubs.net) and TMB scores and MSI scores were calculated.

### Gene Set Enrichment Analysis

To understand the involved biological functions and pathways of PRDM1, gene set enrichment analysis (GSEA) was performed on PRDM1. We employed the molecular signatures Database (MSigDB) H (hallmark gene sets) collection of chemical and genetic perturbations and KEGG subsets of canonical pathways and cancer modules, and the analysis was completed on Sangerbox (http://sangerbox.com/). GSEA results were shown using normalized enrichment scores (NES), accounting for the size and degree to which a gene set is overrepresented at the top or bottom of the ranked list of genes (nominal *p*-value < 0.05 and FDR ≤0.25). To compare the GSEA results between cancers that PRDM1 harbor favorable prognosis and those with unfavorable prognosis, GSEA was conducted separately in LGG, PAAD, UVM, KIRC, SKCM, THCA. The enrichment maps of results were visualized by Bioconductor (http://bioconductor.org/) and R software (http:///www.r-project.org/).

### PRDM1 and Drug Response

The correlation between PRDM1 expression and Drug response was predicted by CellMiner (http://discover.nci.nih.gov/cellminer/).

### Statistical Analysis

In the present work, the clinical survival types, including OS was selected for analysis. Generally, OS is deemed as the duration from the date of diagnosis to the date of death due to any cause.

Wilcox log rank test was adopted to determine the presence or absence of a markedly increased sum of gene expression z-scores in cancer tissues compared with adjacent normal tissues. Meanwhile, Kruskal-Wallis test was employed to compare the difference in the expression of PRDM1. Survival was analyzed by the KM curves, Log-rank test, and the Cox proportional hazards regression model. Spearman test was utilized for correlation analysis. The R language (version 3.6.0; R Foundation) was used for all analyses. A two sided difference of *p* < 0.05 indicated statistical significance.

## Results

### Expression Landscape of the PRDM1

**FIGURE 1:**
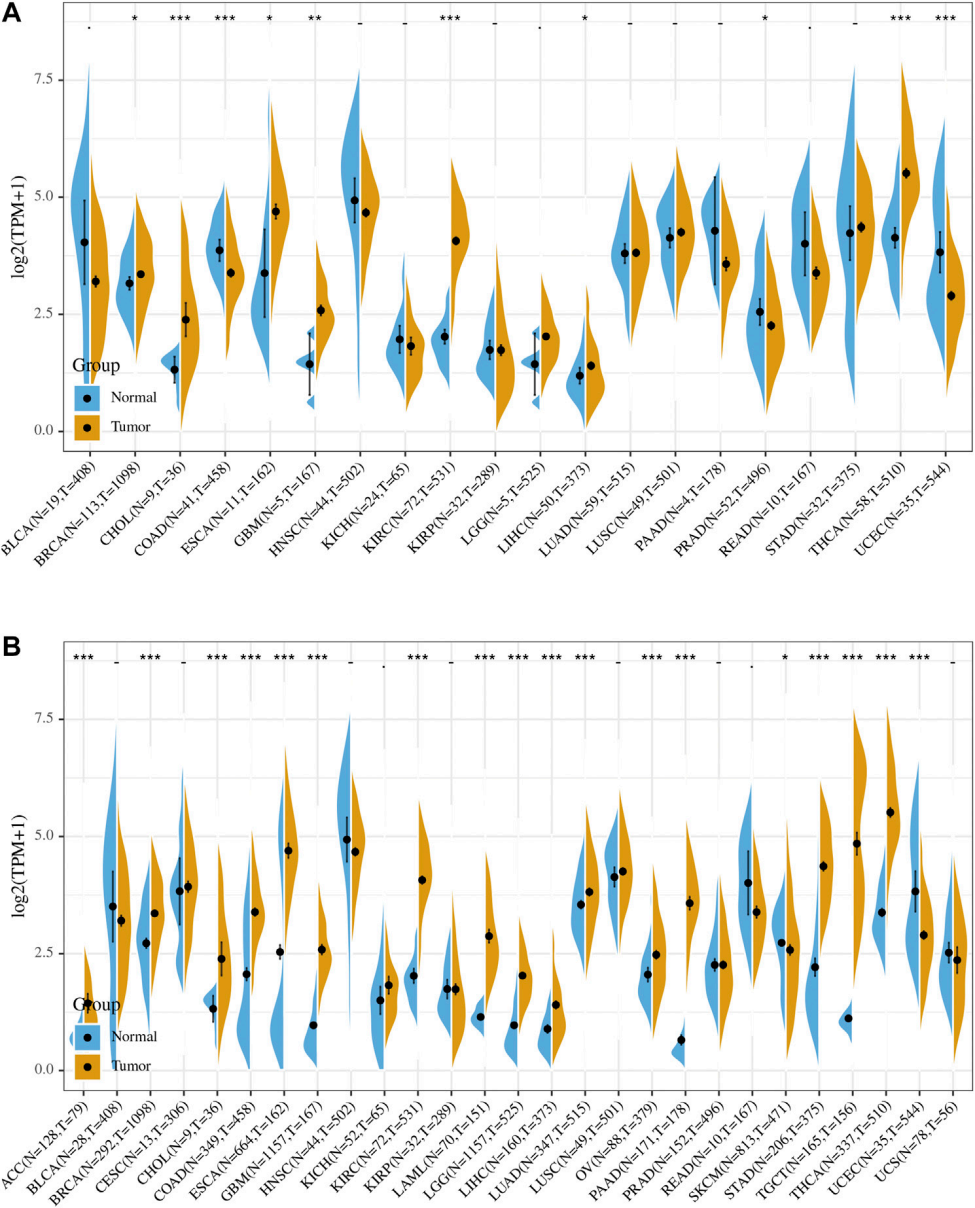
PRDM1 expression levels in different types of human cancers. The expression level of PRDM1 between tumor and normal tissues were compared in twenty cancer types based on the TCGA database **(A)** and twenty-seven cancer types based on the integrated database from TCGA and GTEx datasets **(B)**. Consistent high expression level of PRDM1 were observed in tumor samples of BRCA, CHOL, ESCA, GBM, KIRC, LIHC, THCA than normal tissue based on both comparisons, and significant increased expression of PRDM1 were also seen in ACC, COAD, LAML, LGG, LUAD, OV, PAAD, STAD, TGCT based on the integrated database.

Comparison of expression of PRDM1 between normal and tumor samples across TCGA cancer types and the combined datasets based on integrated database of GTEx and TCGA datasets were conducted and showed in Figure 1. Consistent high expression level of PRDM1 were observed in tumor samples of BRCA, CHOL, ESCA, GBM, KIRC, LIHC, THCA than normal tissue based on both comparisons, and significant increased expression of PRDM1 were also seen in ACC, COAD, LAML, LGG, LUAD, OV, PAAD, STAD, TGCT based on the integrated database. For patients with UCEC, the expression level of PRDM1 was lower in tumor samples compared with normal samples. Patients with different tumor stage and gender didn’t differ in the expression of PRDM1 in tumor samples.

### Correlation of PRDM1 Expression Level and Overall Survival of Cancer Patients

**FIGURE 2:**
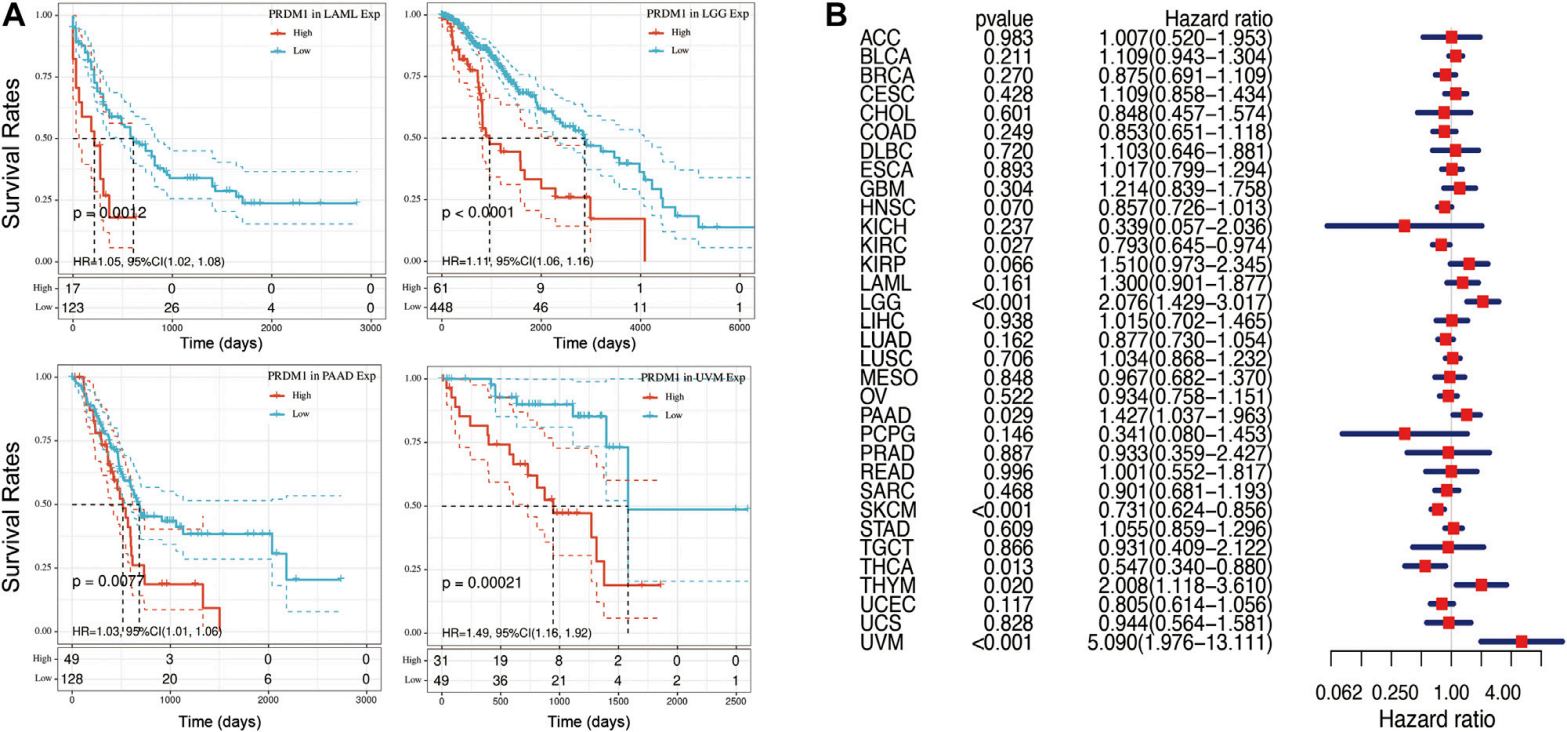
Selected Kaplan-Meier plots and forest plot comparing the high and low expression of PRDN1 on overall survival across different cancers **(A)** Kaplan-Meier Method showed high expression of PRDM1 correlated with unfavorable prognosis in LAML, LGG, PAAD, UVM **(B)** Forest plot displaying the impact of high expression of PRDM1 on OS across thirty three cancer types using Cox regression model.

Figure 2 summarized the results of overall survival (OS) analyses of PRDM1 expression across the 33 cancer types. In univariate analysis, high expression of PRDM1 in tumor samples correlates with poor prognosis in LAML (*p* = 0.001), LGG (*p* < 0.001), PAAD (*p* = 0.008), UVM (*p* < 0.001) (Figure 2A); while high expression of PRDM1 correlates with favorable prognosis in KIRC (*p* = 0.002), SKCM (*p* < 0.001) and THCA (*p* = 0.033). Cox regression model confirmed the prognostic impact of PRDM1 in LGG (HR, 2.08, 95% CI: 1.43–3.02), PAAD (HR, 1.43, 95% CI: 1.04–1.96), UVM (HR, 5.09, 95% CI: 1.98–13.11), KIRC (HR, 0.79, 95% CI: 0.65–0.97), SKCM (HR, 0.73, 95% CI: 0.62–0.86), THCA (HR, 0.55, 95% CI: 0.34–0.88) with the same trend (Figure 2B).

### Correlation of PRDM1 Expression Level and Immune Infiltrates

**FIGURE 3:**
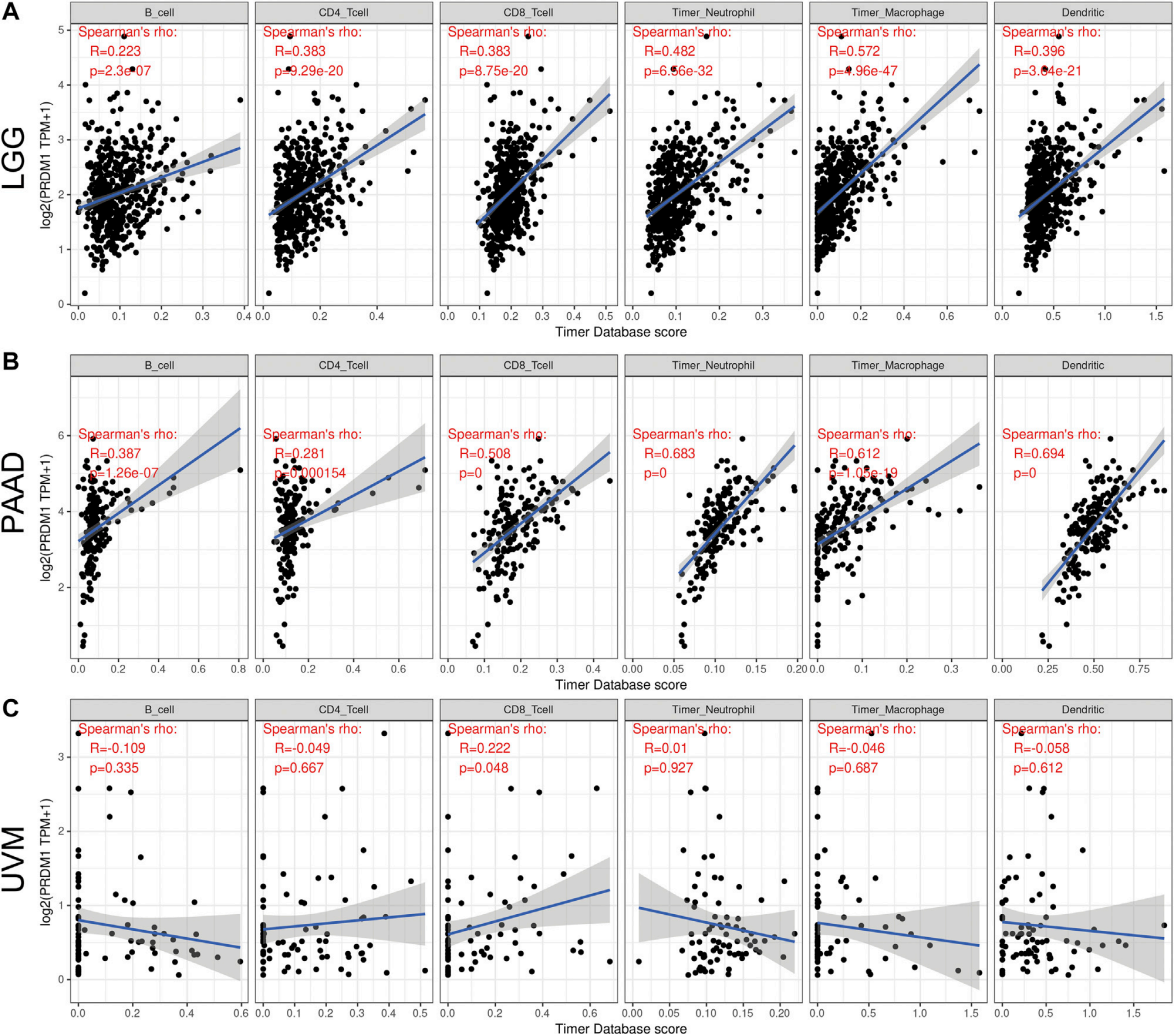
Correlation of PRDM1 expression with immune infiltration level in low grade glioma (LGG), pancreatic adenocarcinoma (PAAD) and uveal melanoma (UVM) **(A and B)** PRDM1 expression is significantly positively correlated with B cell, CD4 T cell, CD8 T cell, Neutrophil, Macrophage and Dendritic Cell infiltration in LGG and PAAD **(C)** PRDM1 expression is significantly positively correlated with CD8 T cell in UVM.

As there are ample evidence supporting the regulatory role of PRDM1, we analyzed its impact on the abundance of immune infiltrates in cancers that harbor prognostic value. TIMER showed PRDM1 positively correlated with the abundance of CD8^+^ T cell in the all cancer types with prognostic significance while it also positively correlated with the abundance B cell, CD4^+^ T cell, Neutrophil, Macrophage and Dendritic cell in all the other cancer types except UVM (Figure 3).

We further calculated the detailed immunocyte compositions of patients in these cancer types through CIBERSORT to explore the key cell type that PRDM1 played a pivotal role (Supplementary Table S1). No individual correlation between the expression of PRDM1 and the abundance of any of the twenty-two immune cell types could perfectly explain the distinct prognostic value of PRDM1 in these cancers.

**FIGURE 4:**
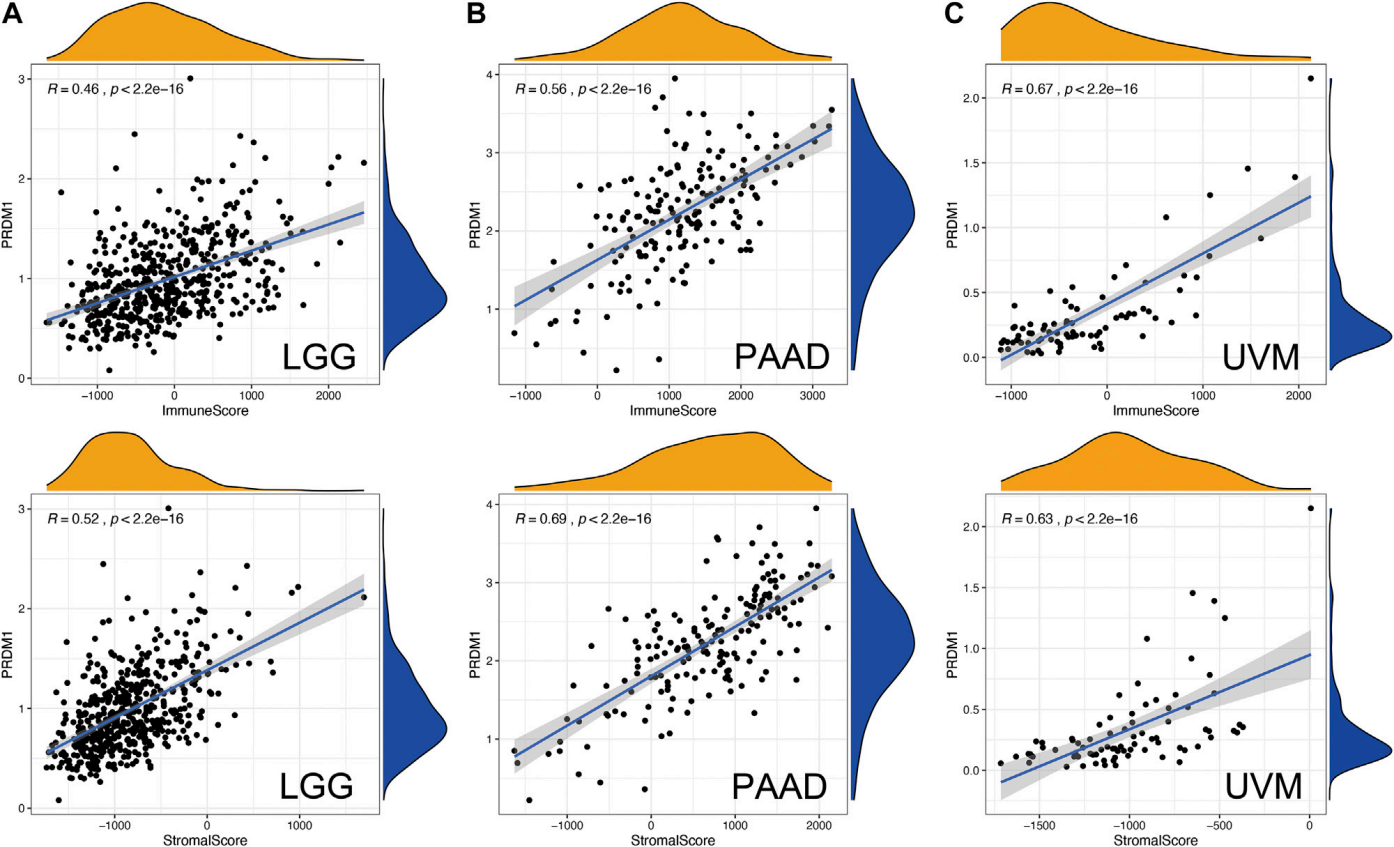
Correlation of PRDM1 expression with Estimate Score in in low grade glioma (LGG), pancreatic adenocarcinoma (PAAD) and uveal melanoma (UVM).

The ESTIMATE method is developed to calculate the immune and stromal scores of cancer tissues. By adopting the ESTIMATE method, we calculated the immune, stromal scores, respectively. The PRDM1 expression was significantly and positively correlated with the stromal, immune scores in both favorable and unfavorable cancers (Figure 4).

### Correlation Analysis on Checkpoint Gene Markers, Tumor Mutation Burden, Microsatellite Instability

**FIGURE 5:**
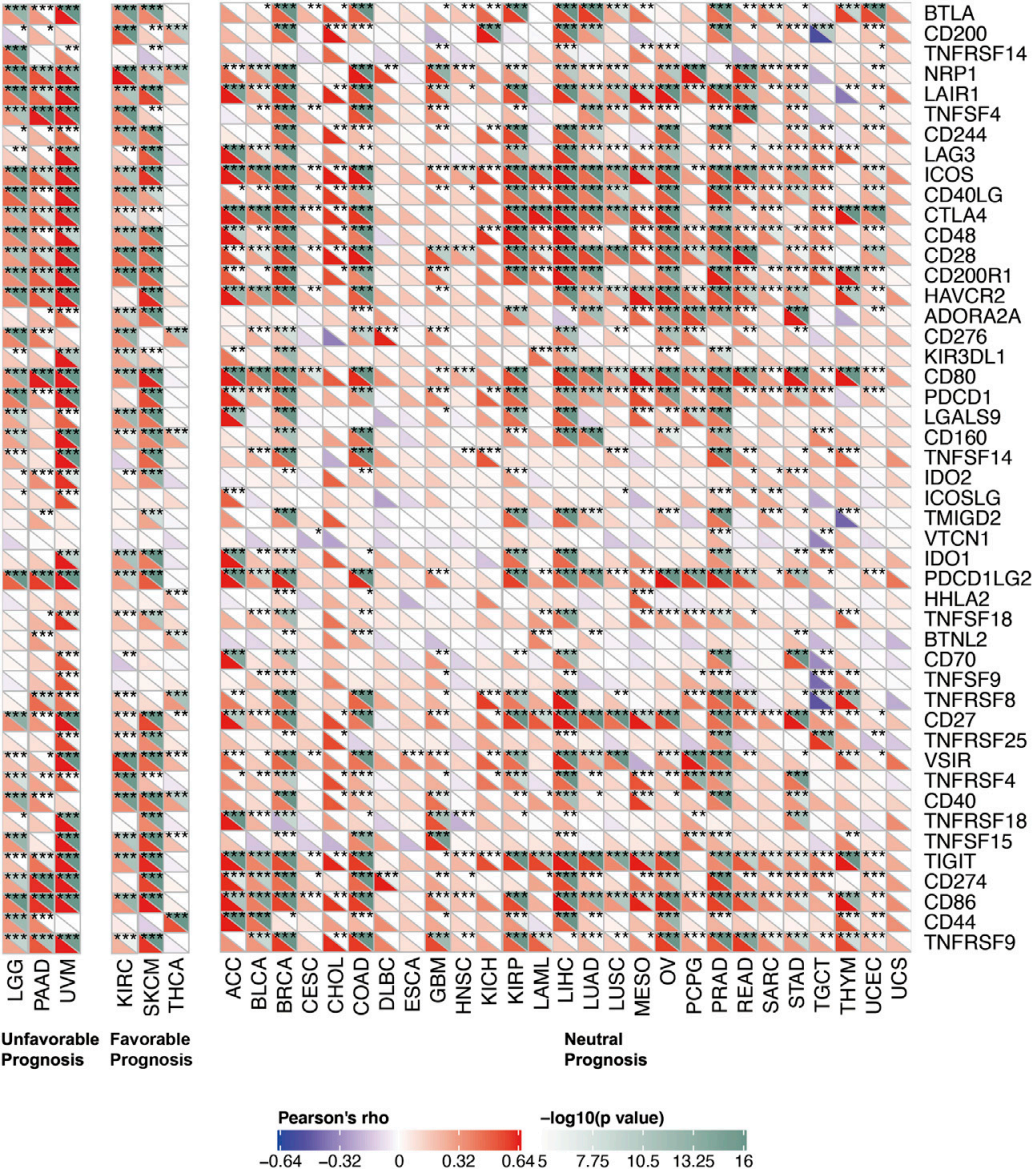
Correlation of PRDM1 expression with expression of immune checkpoint genes across 33 cancer types.

**FIGURE 6:**
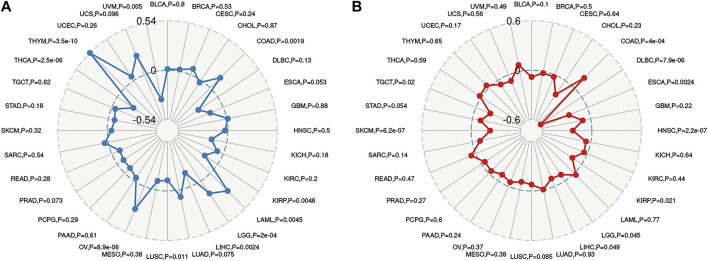
Radar map plotting the correlations between PRDM1 expression and Tumor mutation burden **(A)**, as well as the correlations between PRDM1 expression and microsatellite instability **(B)** across 33 cancer types.

To further explored the potential mechanism of immune inhibition of PRDM1 signaling, the associations of PRDM1 expression with multiple checkpoint markers were compared across different cancer types (Figure 5). Generally, PRDM1 expression positively correlates with the expression of LAG3, CTLA4, PDCD1 (PD-1), CD274 (PD-L1), PDCD1LG2 (PD-L2), TIGIT in the majority of 33 cancer types. It is notable that PRDM1 seemed to positively correlated with TNFRSF14 in LGG and UVM among cancers with unfavorable prognosis, while this correlation seems weak or even negative in all cancers with favorable prognosis. It is also important that strong and positive correlations between PRDM1 and PD1 were seen in all the unfavorable cancer types while this correlation is only present in SKCM among all cancer types with favorable prognosis. Moreover, we evaluated the association of TMB/MSI with the PRDM1 expression, as shown in Figure 6. Expression of PRDM1 positively correlated with the TMB in THYM, LAML, LGG, COAD while negatively correlated with the TMB in LIHC, LUSC, THCA, UVM (Figure 6A). Expression of PRDM1 negatively correlated with MSI in DLBC, TGCT, SKCM, LIHC, KIRP, LGG, HNSC, ESCA while positively correlated with it in COAD (Figure 6B).

### Functional Analysis by Gene Set Enrichment Analysis

**FIGURE 7:**
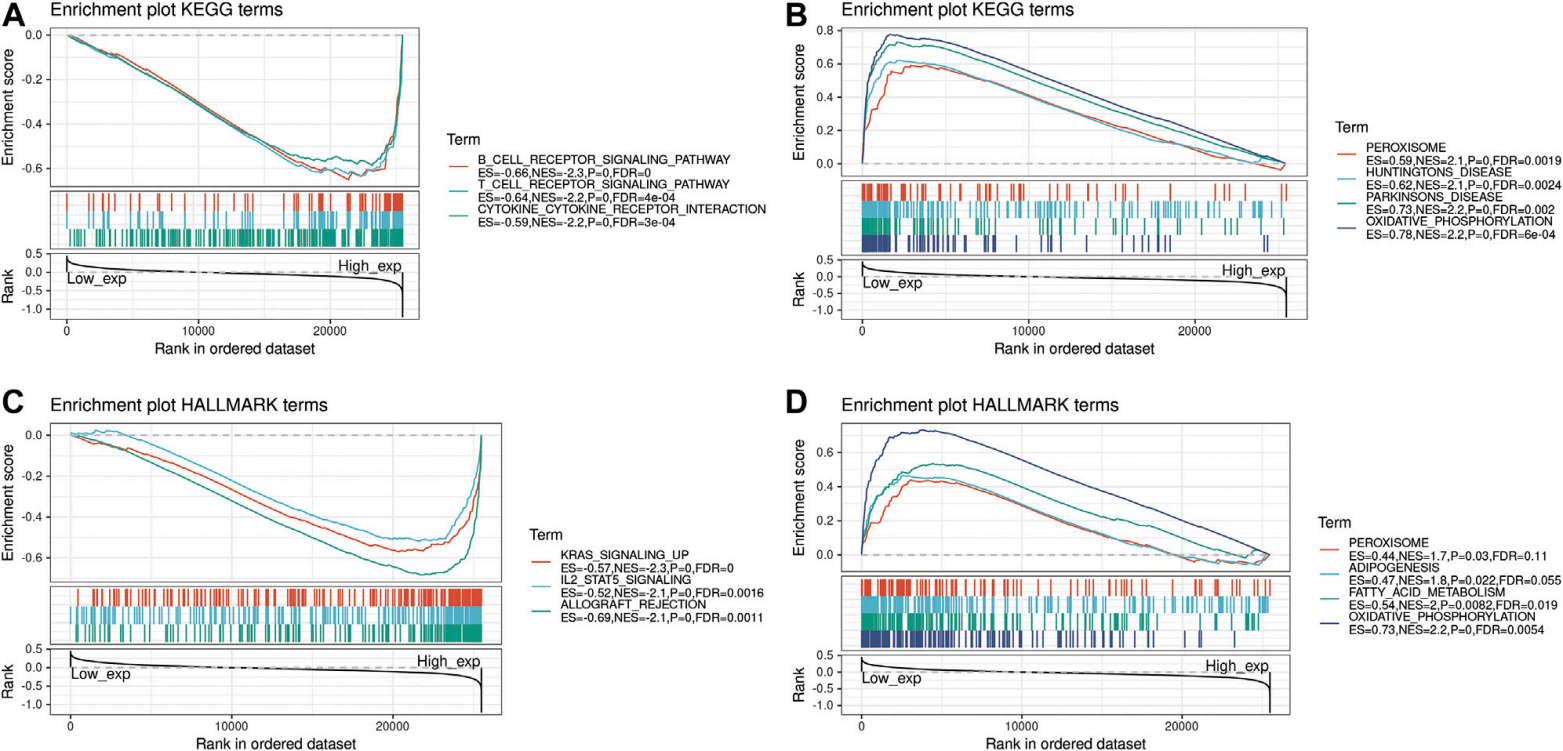
Functional Enrichment of KEGG and HALLMARK terms on PRDM1 through GSEA. The top three negative and positive enriched KEGG terms were displayed in **(A)** and **(B)** respectively. The top three negative and positive enriched HALLMARK terms were displayed in **(C)** and **(D)** respectively.

The biological role of the PRDM1 was explored through GSEA. The pan-cancer functional KEGG and HALLMARK terms of the PRDM1 are listed in Supplementary Table S2. Generally, the top three negatively enriched KEGG terms in high PRDM1 subgroup were B cell receptor signaling, T cell receptor signaling and cytokine cytokine receptor interaction (Figure 7A) and the top negatively enriched HALLMARK terms included IL-2-STAT5 signaling and allograft rejection (Figure 7B). The top positively enriched terms included peroxisome and oxidative phosphorylation (Figure 7B,D). Further comparison of results among selected cancers showed that the GO term negative regulation of cell activation was enriched in PAAD, and GO term regulation of lymphocyte activation was enriched in UVM, while these two terms were absent in cancers with favorable prognosis (Supplementary Table S3).

### PRDM1, Cancer Stemness and Drug Response

**FIGURE 8:**
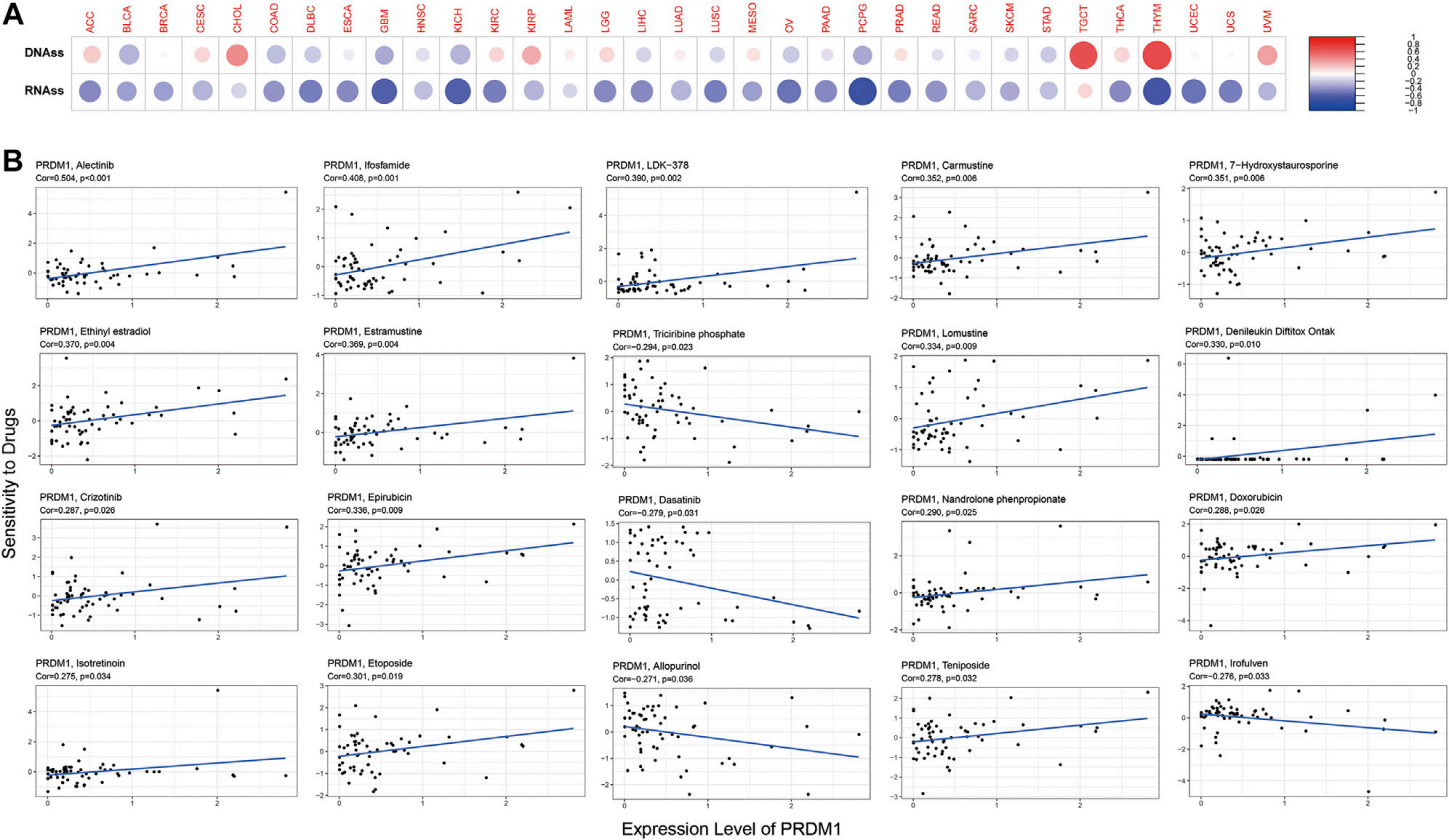
PRDM1 expression, cancer stemness and drug response. PRDM1 expression was found to be positively correlated with cancer stemness in CHOL, KIRP, TGCT, THYM and UVM (Figure 8A). The correlation between PRDM1 expression and predicted drug response was displayed in Figure 8B.

The expression of PRDM1 positively correlated with cancer stemness in CHOL, KIRP, TGCT, THYM and UVM (Figure 8A). The correlation between PRDM1 expression and predicted drug response was displayed in Figure 8B. Moreover, we evaluated the association of TMB/MSI with the PRDM1 expression, as shown in Figure 6 and Supplementary Figure S1.

## Discussion

The present work illustrated a comprehensive workflow for pan-cancer analysis and thoroughly investigated the role of the PRDM1 in cancers. The results showed the prognostic impact of PRDM1 across the different cancer types. PRDM1 expression positively correlates with the expression of LAG3, CTLA4, PDCD1 (PD-1), CD274 (PD-L1), PDCD1LG2 (PD-L2), TIGIT in the majority of 33 cancer types, and GSEA demonstrated the high PRDM1 patient group negatively enriched terms including B cell receptor signaling, T cell receptor signaling and cytokine cytokine receptor interaction, IL-2-STAT5 signaling and allograft rejection.

Our study showed that PRDM1 harbor distinct prognostic values across different cancer types. Generally, PRDM1/BLIMP1 play an immune suppressive role in modulating the local tumor microenvironment. PRDM1 regulates the responsiveness and homeostasis of peripheral T cells by attenuating both proliferation and survival (Boi et al., 2015). T cell activation induces IL-2 transcription, IL-2 signaling, in turn, induces PRDM1 transcription, and BLIMP1 feeds back to induce IL-10 transcription and repress IL-2 transcription. In addition, high levels of BLIMP1 in Treg cells and the inflammatory disease associated with BLIMP1 ablation in Treg cells suggest a role of this transcription factor in Treg cell differentiation and/or function. On the hand, there are also reported showed PRDM1 in cancer cell plays a tumor suppressive role. One study in lung cancer showed downregulation of PRDM1 *in vitro* promotes cellular invasion and metastasis (Zhu et al., 2017). Liu et al. found forced expression of PRDM1 in human colon cancer organoids prevents the formation and growth of colon tumor organoids *in vitro* (Liu et al., 2018). Wang et al. reported that PRDM1 attenuated glioma malignancy by negatively modulating Wnt/b-catenin signaling and this modulation was dependent on the Wnt inhibitor Dkk1 (Wang et al., 2013). Therefore, the distinct prognostic value of high PRDM1 expression across different cancers may result from its synthetic effect of immune suppressive activity and tumor suppressive activity in each cancer type. This could also be explained by the GSEA results in our study that cancers that PRDM1 harbor unfavorable prognosis identified enriched GO terms of negative regulation of cell activation and regulation of lymphocyte activation that not found in cancers with favorable prognosis.

The PRDM1 expression was correlated with TMB and MSI in some cancer types. TMB affects the generation possibility of immunogenic peptide, thus impacting the patient response to immune checkpoint inhibitors (Chan et al., 2019). MSI is a vital index to predict tumor genesis and development (Chang et al., 2018). The NCCN guidelines have recommended MSI testing for all colorectal cancer (READ) subtypes, and the READ mortality can be reduced by the early detection of MSI (Ganesh et al., 2019). FDA has approved the use of Keytruda for the treatment of MSI-H solid tumors. Therefore, TMB and MSI can serve as the predicting factors for the efficacy. Our findings provide clues on the correlation between the expression of PRDM1 and cancer immunity and suggests that it could be a potential predictive maker of the efficacy of immunotherapy.

Targeting PRDM1/BLIMP1 could be a promising immunotherapeutic strategy in the management of LGG, PAAD and UVM in the future (Li et al., 2019). Currently, there are no small molecule drugs specifically targeting PRDM1 available. Effort are needed to develop novel drugs or RNAi techniques targeting PRDM1 specifically in tumoral infiltrative immune cells. On the other hand, engineering tumor specific T cells by down regulating the expression of PRDM1 could also be a promising strategy to enhance the efficacy of adoptive immunotherapy.

Our study has several limitations. First, the mRNA level of PRDM1 is assessed in our study, while its correlation with the amount of translational product needed to be validate in future experiments.

Secondly, the result of our study lacks external validation in other public datasets. More efforts are needed to undermine the value of PRDM1 as a potential target of immunotherapy.

## Data Availability Statement

Publicly available datasets were analyzed in this study. The data can be found here: The TCGA project (https://portal.gdc.cancer.gov).

## Ethics Statement

Ethical review and approval was not required for the study on human participants in accordance with the local legislation and institutional requirements. Written informed consent for participation was not required for this study in accordance with the national legislation and the institutional requirements.

## Funding

This study was supported by National Natural Science Foundation of China, Young Scientists Fund (81801804).

## Author Contributions

LS and WF designed and implemented the study design. LS, QC, CY, YW, SC, HY, SO, YJ, YW, TH, LK, JM, ZF and PZ participated in data analysis. ZF, and PZ managed and advised on the project. LS, QC, CY, YW, SC, and HY wrote the paper.

## Conflict of Interest

The authors declare that the research was conducted in the absence of any commercial or financial relationships that could be construed as a potential conflict of interest.

## References

[B1] Becht E. Giraldo N. A. Lacroix L. Buttard B. Elarouci N. Petitprez F. (2016). Estimating the population abundance of tissue-infiltrating immune and stromal cell populations using gene expression. Genome Biol. 17 (1) 218 10.1186/s13059-016-1070-5 27765066 PMC5073889

[B2] Boi M. Zucca E. Inghirami G. Bertoni F. (2015). PRDM1/BLIMP1: a tumor suppressor gene in B and T cell lymphomas. Leuk. Lymphoma 56 (5), 1223–1228. 10.3109/10428194.2014.953155 25115512

[B3] Chan T. A. Yarchoan M. Jaffee E. Swanton C. Quezada S. A. Stenzinger A. (2019). Development of tumor mutation burden as an immunotherapy biomarker: utility for the oncology clinic. Ann. Oncol. 30 (1), 44–56. 10.1093/annonc/mdy495 30395155 PMC6336005

[B4] Chang L. Chang M. Chang H. M. Chang F. (2018). Microsatellite instability: a predictive biomarker for cancer immunotherapy. Appl. Immunohistochem. Mol. Morphol. 26 (2), e15–e21. 10.1097/PAI.0000000000000575 28877075

[B5] Chihara N. Madi A. Kondo T. Zhang H. Acharya N. Singer M. (2018). Induction and transcriptional regulation of the co-inhibitory gene module in T cells. Nature 558 (7710), 454–459. 10.1038/s41586-018-0206-z 29899446 PMC6130914

[B6] Cristescu R. Mogg R. Ayers M. Albright A. Murphy E. Yearley J. (2018). Pan-tumor genomic biomarkers for PD-1 checkpoint blockade-based immunotherapy. Science 362 (6411), eaar3593 10.1126/science.aar3593 30309915 PMC6718162

[B7] Fu S. H. Yeh L. T. Chu C. C. Yen B. L. Sytwu H. K. (2017). New insights into Blimp-1 in T lymphocytes: a divergent regulator of cell destiny and effector function. J. Biomed. Sci. 24 (1), 49 10.1186/s12929-017-0354-8 28732506 PMC5520377

[B8] Ganesh K. Stadler Z. K. Cercek A. Mendelsohn R. B. Shia J. Segal N. H. (2019). Immunotherapy in colorectal cancer: rationale, challenges and potential. Nat. Rev. Gastroenterol. Hepatol. 16 (6), 361–375. 10.1038/s41575-019-0126-x 30886395 PMC7295073

[B9] Li N. Fan X. Wang X. Deng H. Zhang K. Zhang X. (2019). PRDM1 levels are associated with clinical diseases in chronic HBV infection and survival of patients with HBV-related hepatocellular carcinoma. Int. Immunopharm. 73, 156–162. 10.1016/j.intimp.2019.05.012 31100710

[B10] Li T. Fan J. Wang B. Traugh N. Chen Q. Liu J. S. (2017). TIMER: a web server for comprehensive analysis of tumor-infiltrating immune cells. Canc. Res. 77 (21), e108–e10. 10.1158/0008-5472.CAN-17-0307 PMC6042652 29092952

[B11] Liu C. Banister C. E. Weige C. C. Altomare D. Richardson J. H. Contreras C. M. (2018). PRDM1 silences stem cell-related genes and inhibits proliferation of human colon tumor organoids. Proc. Natl. Acad. Sci. USA. 115 (22), E5066–E75. 10.1073/pnas.1802902115 29760071 PMC5984534

[B12] Liu G. S. Wang J. P. Chu Z. H. Yu Y. Y. Zhou W. (2017). Effect of B Lymphocyte-Induced mature protein-1 expression in bone marrow mononuclear cells on prognosis of patients with multiple myeloma. Zhongguo Shi Yan Xue Ye Xue Za Zhi 25 (5), 1449–1453. 10.7534/j.issn.1009-2137.2017.05.029 29070123

[B13] Martins G. A. Cimmino L. Shapiro-Shelef M. Szabolcs M. Herron A. Magnusdottir E. (2006). Transcriptional repressor Blimp-1 regulates T cell homeostasis and function. Nat. Immunol. 7 (5), 457–465. 10.1038/ni1320 16565721

[B14] Newman A. M. Steen C. B. Liu C. L. Gentles A. J. Chaudhuri A. A. Scherer F. (2019). Determining cell type abundance and expression from bulk tissues with digital cytometry. Nat. Biotechnol. 37 (7), 773–782. 10.1038/s41587-019-0114-2 31061481 PMC6610714

[B15] Nutt S. L. Fairfax K. A. Kallies A. (2007). BLIMP1 guides the fate of effector B and T cells. Nat. Rev. Immunol. 7 (12), 923–927. 10.1038/nri2204 17965637

[B16] Pan J. H. Zhou H. Cooper L. Huang J. L. Zhu S. B. Zhao X. X. (2019). LAYN is a prognostic biomarker and correlated with immune infiltrates in gastric and colon cancers. Front. Immunol. 10, 6 10.3389/fimmu.2019.00006 30761122 PMC6362421

[B17] Rutz S. Ouyang W. (2016). Regulation of interleukin-10 expression. Adv. Exp. Med. Biol. 941, 89–116. 10.1007/978-94-024-0921-5_5 27734410

[B18] Tomczak K. Czerwinska P. Wiznerowicz M. (2015). The Cancer Genome Atlas (TCGA): an immeasurable source of knowledge. Contemp. Oncol. 19 (1A), A68–A77. 10.5114/wo.2014.47136 PMC4322527 25691825

[B19] Wang X. Wang K. Han L. Zhang A. Shi Z. Zhang K. (2013). PRDM1 is directly targeted by miR-30a-5p and modulates the Wnt/β-catenin pathway in a Dkk1-dependent manner during glioma growth. Canc. Lett. 331 (2), 211–219. 10.1016/j.canlet.2013.01.005 23348703

[B20] Zhu Z. Wang H. Wei Y. Meng F. Liu Z. Zhang Z. (2017). Downregulation of PRDM1 promotes cellular invasion and lung cancer metastasis. Tumour Biol 39 (4), 1010428317695929 10.1177/1010428317695929 28378641

