# Supplementary figures and images for: Role of PRDM1 in Tumor Immunity and Drug Response: A Pan-Cancer Analysis
Source: Front Pharmacol. 2020 Dec 15;11:593195. doi: 10.3389/fphar.2020.593195 (PMC7770985; doi:10.3389/fphar.2020.593195)

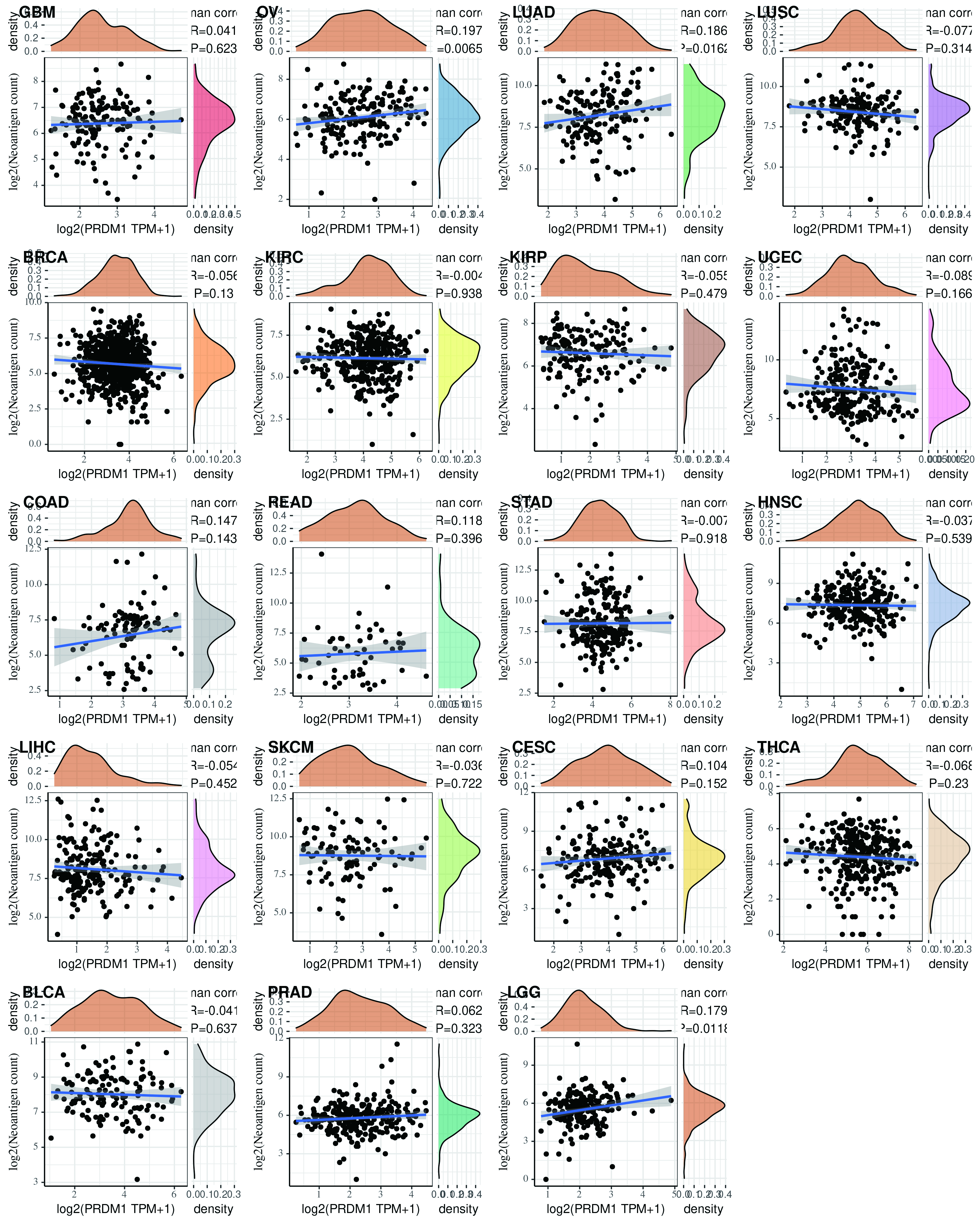

Supplement: Supplementary file 4 [file image1.tif]
